# Effect of Tetramethylpyrazine on Neuroplasticity after Transient Focal Cerebral Ischemia Reperfusion in Rats

**DOI:** 10.1155/2021/1587241

**Published:** 2021-01-18

**Authors:** Junbin Lin, Chizi Hao, Yu Gong, Ying Zhang, Ying Li, Zhihe Feng, Xiangdong Xu, Hailong Huang, Weijing Liao

**Affiliations:** Department of Neurological Rehabilitation, Zhongnan Hospital of Wuhan University, 169 Donghu Road, Wuhan 430071, Hubei, China

## Abstract

Tetramethylpyrazine (TMP) has been widely used in ischemic stroke in China. The regulation of neuroplasticity may underlie the recovery of some neurological functions in ischemic stroke. Middle cerebral artery occlusion (MCAO) model was established in this study. Rats were divided into three groups: sham group, model group, and TMP group. The neurological function was evaluated using modified neurological severity score (mNSS). Following the neurological function test, expression of synaptophysin (SYP) and growth-associated protein 43 (GAP-43) were analyzed through immunohistochemistry at 3 d, 7 d, 14 d, and 28 d after MCAO. Finally, the synaptic structural plasticity was investigated using transmission electron microscopy (TEM). The TMP group showed better neurological function comparing to the model group. SYP levels increased gradually in ischemic penumbra (IP) in the model group and could be enhanced by TMP treatment at 7 d, 14 d, and 28 d, whereas GAP-43 levels increased from 3 d to 7 d and thereafter decreased gradually from 14 d to 28 d in the model group, which showed no significant improvement in the TMP group. The results of TEM showed a flatter synaptic interface, a thinner postsynaptic density (PSD), and a wider synaptic cleft in the model group, and the first two alterations could be ameliorated by TMP. Then, a Pearson's correlation test revealed mNSS markedly correlated with SYP and synaptic ultrastructures. Taken together, TMP is capable of promoting functional outcome after ischemic stroke, and the mechanisms may be partially associated with regulation of neuroplasticity.

## 1. Introduction

Globally, stroke is a leading cause of mortality and disability, and the substantial economic costs for poststroke care and treatment are needed [1]. A study from the Global Burden of Diseases, Injuries, and Risk Factors Study (GBD) showed that stroke is the second largest cause of death worldwide (5.5 million (95% UI 5.3–5.7)) in 2016, and the overall burden of stroke remains high [2]. Of all strokes, approximately 87% are ischemic strokes and 13% are hemorrhagic [3]. Ischemic stroke is compromised by reduction in blood flow and subsequent infarction which resulted from atherosclerotic or thromboembolic events [2]. For ischemic stroke, current therapeutic management did not show fully satisfactory outcomes despite decades of extensive efforts in study of stroke mechanisms and therapeutic interventions. Up to now, intravenous thrombolysis with recombinant tissue plasminogen activator (rt-PA) remained the only pharmacological treatment approved by the US Food and Drug Administration (FDA) for ischemic stroke patients [4]. However, because of limited therapeutic time window (within 4.5 h) and potential side effect of intracranial hemorrhage, the use of rt-PA was strictly restricted to few stroke patients [5]. Therefore, new therapies were needed to protect and repair the damaged brain after stroke.

**Figure 1:**
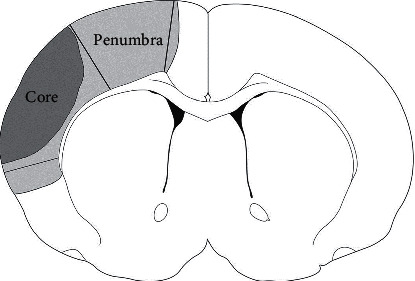
Schematic presentation of IP and ischemic core.

Ischemic penumbra (IP), located in the peri-infarct area (Figure 1) [6], is an important region of reduced cerebral blood flow (CBF) with functional impairment and electrophysiologic disturbances which still have the potential to recover if perfusion is improved [7]. Neuroplasticity is an important process in IP and considered to be a therapeutic target of ischemic stroke [8].

**Figure 2:**
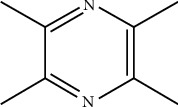
The chemical structure of TMP.

Brain could compensate damages through functional reorganization and reestablishment of new connections among undamaged neurons [9]. In this process, structural changes may underpin the neural plasticity and functional recovery [10]. After stroke, a series of spontaneous repair, including expression of key growth factors, growth of synapses and dendrites, axonal remodeling, and angiogenesis, occurred and could be promoted by treatments [11]. 2,3,5,6-tetramethylpyrazine (TMP, Figure 2), also known as ligustrazine, is an important bioactive ingredient extracted from Chinese herbal medicine *Ligusticum wallichii Franchat* (Chuan Xiong) and has been widely used in ischemic stroke by Chinese doctors [12]. Experimental studies have proved that TMP could regulate cerebral plasticity via promoting cell proliferation, differentiation [13], and migration [14]. In addition, we previously reported that TMP could increase the expression of microtubule-associated protein-2 (MAP-2) and enhance dendritic plasticity in an experimental stroke model [15]. In this present study, we hypothesized that TMP would contribute to promotion of neuroplasticity. Focal cerebral ischemia reperfusion was induced by middle cerebral artery occlusion (MCAO) in rats, and we evaluated the effects of TMP on neurological function. At different time points, we studied the expression of synaptophysin (SYP, marker of synaptogenesis) and growth-associated protein 43 (GAP-43, marker of axonal regeneration and sprouting). Then, we also investigated the synaptic structural plasticity using transmission electron microscopy (TEM). Finally, correlations between neurological function and other results were performed using Pearson's correlation test.

## 2. Materials and Methods

### 2.1. Animals

Eight-week-old male Sprague Dawley (SD) rats weighing 200–250 g (purchased from Experimental Animal Center of Wuhan University) were used for this experiment. The rats were acclimated for 3 or more days before the start of any experiments. They were housed in a controlled environment (four animals per cage, 55 ± 5% relative humidity, 22°C, 12 : 12 h light/dark cycle) and provided with free access to food and water. All experimental procedures involving animals were conducted in strict accordance with the guidelines approved by the Animal Care and Use Committee of Wuhan University Medical School. We made all efforts to minimize the number of animals used and their suffering.

### 2.2. Establishment of Model

Transient focal cerebral ischemia was induced by MCAO using the intraluminal filament technique with some modifications [16]. The rats were anesthetized with sodium pentobarbital (50 mg/kg) intraperitoneally, and after a median incision of the neck skin, the right common carotid artery (CCA), external carotid artery (ECA), and internal carotid artery (ICA) were carefully isolated. The right middle cerebral artery (MCA) was occluded with a monofilament nylon filament (Beijing Cinonteh Biotech Co, Beijing, China) by inserting it through the right CCA and gently advancing into the ICA up to a point approximately 17 mm distal to the bifurcation of the CCA. The filament was fixed in place, and the animal was allowed to recover from anesthesia. Reperfusion was performed by withdrawing the filament by about 10 mm after 120 min without anesthesia. A heating pad was used to maintain a rectal temperature of 37.0 ± 0.5°C during the surgical procedure. Sham-operated rats underwent the same procedure without inserting a nylon filament.

### 2.3. Grouping and Administration

**Figure 3:**
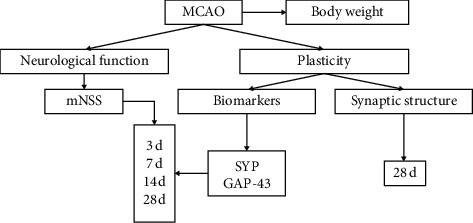
A brief flowchart of experimental protocol.

The animals were randomly assigned into 3 groups: sham group, model group, and TMP group (*n* = 10 per group). In the TMP group, animals received 20 mg/kg TMP (Aladdin Chemistry Co, Shanghai, China) intraperitoneally immediately after reperfusion. In the sham and control groups, animals were served with injection of the same volume of saline at the same time. All injections were conducted through intraperitoneal injection daily and in volume of 5 ml/kg until the day before sacrificing. The animals were sacrificed at 3, 7, 14, and 28 d after MCAO for immunohistochemistry and 28 d for TEM.  Figure 3 shows a brief flowchart of our study.

### 2.4. Neurological Functional Test

A modified neurological severity score (mNSS) test [17] was employed to evaluated neurological function (*n* = 10). It was performed at 3 d, 7 d, 14 d, and 28 d after MCAO in a blind fashion by another investigator. MNSS is a composite of motor, sensory, reflex, and balance tests and graded on a scale of 0–18 (normal score 0, maximal deficit score 18). A higher score means more severe injury.

### 2.5. Immunohistochemistry

At 3 d, 7 d, 14 d, and 28 d after MCAO, the rats in each group (*n* = 6) were anesthetized and perfused transcardially with 150 ml of 0.9% saline and 150 ml of 4% paraformaldehyde. The brain was removed and postfixed in 4% paraformaldehyde for 24 h at room temperature. Thereafter, the brain was cut into 4 mm thick coronal blocks (bregma: −2 to +2 mm). After embedded by paraffin, the sections of 6 *μ*m were mounted onto the polylysine-coated slides. The immunohistochemical staining was performed using the streptavidin-peroxidase method [18] as follows: the tissue sections were dewaxed with xylene and rehydrated in ethanol. Then, the specimens were incubated in endogenous peroxidase blocking solution (Maixin Technology Co, Fuzhou, Fujian, China) for 10 min. After incubated with normal rabbit serum (Maixin Technology Co, Fuzhou, Fujian, China), the mouse anti-SYP primary antibody (1 : 200, Boster, Wuhan, Hubei, China) and mouse anti-GAP-43 primary antibody (1 : 200, Boster, Wuhan, Hubei, China) were added and incubated, respectively, with samples at 4°C overnight. A biotin-conjugated secondary antibody (Maixin Technology Co, Fuzhou, Fujian, China) was added to the samples and incubated at room temperature for 15 min. Then, each section was incubated with HRP-streptavidin-peroxidase (Maixin Technology Co, Fuzhou, China) for 15 min. Next, each section was treated with 3,3′-diaminobenzidine (DBA) and counterstained with hematoxylin. Between every procedure, the sections were rinsed with phosphate-buffered saline (PBS, pH = 7.4) 3 times for 3 min. Finally, the sections were dehydrated and cover-slipped. Negative controls were established by replacing the primary antibody with PBS and normal rabbit serum.

Another investigator evaluated immunohistochemical results blindly. Six randomly selected sections (three for GAP-43 and three for SYP) of each subject were used for statistic analysis. 5 arbitrary fields (40 × objective) from each section in IP of hemisphere with the lesion were randomly captured using a digital camera. Image Pro Plus 6.0 (IPP 6.0, Media Cybernetics Inc, Bethesda, MD, USA) was used to measure integral optical density (IOD) of every photographs.

### 2.6. Transmission Electron Microscopy

**Figure 4:**
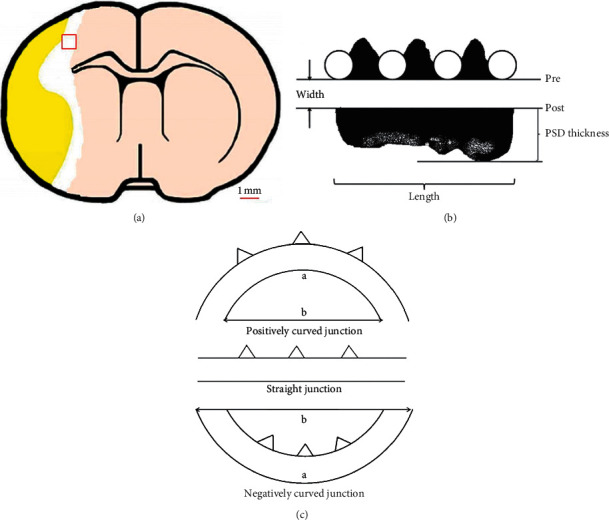
Sample collection and measurement of synaptic ultrastructures. (a) 1  mm × 1  mm × 1 mm cerebral parietal cortex tissue samples were collected from IP. (b) Schematic diagram of how to measure the thickness of PSD, width of synaptic cleft, and the length of active zone. (c) Three types of synaptic interface (positively curved, straight, and negatively curved junction). The curvature of straight synapse is 1.00, and a/b is the curvature of the other two types of synapses.

At 28 d after MCAO, the rats in each group (*n* = 4) were anesthetized and perfused transcardially with 250 ml 4% paraformaldehyde. 1 mm × 1 mm × 1 mm tissue samples were collected from parietal cortex of IP as quickly as possible (Figure 4(a)) and fixed in 2.5% phosphate-buffered glutaraldehyde for 24 h. Then, the samples were prepared for TEM routinely with some modifications [19]: (1) the samples were rinsed three times with 0.1 M PBS and postfixed in 1% OsO_4_ for 2 h in 4°C; (2) after rinsed three times with 0.1 M PBS, they were dehydrated with ethanol and acetone in a graded series; (3) they were embedded in a 1 : 1 mixture of Epon 812 and acetone for 30 min; (4) the blocks were placed in 37°C for 24 h and then 60°C for 48 h; (5) the ultrathin sections (approximately 60 nm thick) were cut using an ultramicrotome (LKB-V, LKB Produkter AB, Sweden) and stained with uranyl acetate and lead citrate for 10 min each.

The sections were viewed at × 10000 under a HT-7700 TEM (Hitachi, Japan). In this study, the ultrastructures of synapses were evaluated using IPP 6.0 program. The parameters contained curvature of synaptic interface [20], thickness of postsynaptic density (PSD) [21], width of synaptic cleft (using multipoint average method), and length of the active zone [21] (Figures 4(b) and 4(c)). Totally, 40 micrographs (10 per animal) of each group were taken for measurement.

### 2.7. Statistical Analysis

All data were expressed as mean ± standard deviation (SD). Statistical analysis was performed by using the GraphPad Prism 8.3.0 (GraphPad Software, La Jolla, CA, USA). Immunohistochemistry data were analyzed using linear mixed-effects analyses followed by Tukey's post hoc comparisons. The data of mNSS were also analyzed using linear mixed-effects analyses while followed by Sidak's post hoc comparisons (model vs. TMP). The Greenhouse–Geisser correction was applied if the assumptions of sphericity were violated. TEM data were analyzed using one-way ANOVA followed by Tukey's post hoc comparisons. Correlation analysis between mNSS and other data was evaluated by Pearson's correlation test. A value of *P* value below 0.05 was considered statistically significant.

## 3. Results

### 3.1. Effect of TMP on Neurological Function Recovery

**Figure 5:**
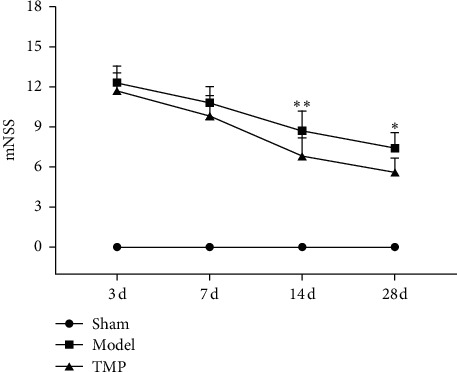
Neurological function by mNSS from 3 d to 28 d after MCAO (mean ± SD, *n* = 10). ^*∗*^ indicates *P* < 0.05, and ^*∗∗*^ indicates *P* < 0.01 (model vs. TMP).

A 18-point mNSS was used to measure the animals' neurological outcome following MCAO (*n* = 10 in every group). All rats in the sham group show 0 score without any neurological function impairment. Linear mixed-effects analyses indicated that there was a significant effect of time (*F*_3, 54_ = 105.67, *P* < 0.001) and intervention (*F*_1, 18_ = 10.01, *P*=0.005) on mNSS but no interaction between them (*F*_3, 54_ = 1.70, *P*=0.177).  Figure 5 shows that the model group and TMP group improved their neurological performance overtime, which displayed a declining curve of mNSS scores. According to the results of multiple comparisons, TMP significantly improved neurological function as evidenced by lower mNSS at 14 d (*P*=0.008) and 28 d (*P*=0.013) after MCAO.

### 3.2. Effect of TMP on Expression of SYP

**Figure 6:**
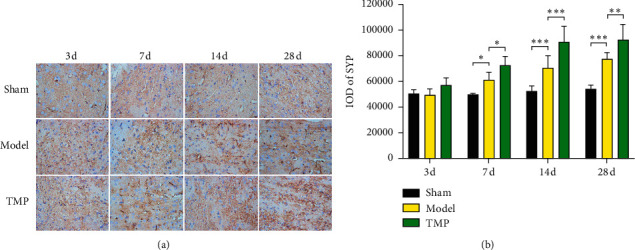
Identification of SYP by immunohistochemical assay (×400) in IP with time going on 3 d, 7 d, 14 d, and 28 d after MCAO. (a) Representative immunohistochemical images of SYP expression. (b) Quantification of the SYP levels through measuring the IOD values (mean ± SD, *n* = 6). ^*∗*^ indicates *P* < 0.05, ^*∗∗*^ indicates *P* < 0.01, and ^*∗∗∗*^ indicates *P* < 0.001.

In this study, IOD values were applied to indicate the expression of SYP and GAP-43. We obtained 15 IODs (5 fields × 3 sections), and the mean value was used for analysis (*n* = 6 in every group). There was a significant effect of time (*F*_3, 60_ = 37.47, *P* < 0.001) and intervention (*F*_2, 60_ = 85.71, *P* < 0.001) on expression of SYP in IP; moreover, a significant interaction effect between time and intervention existed (*F*_6, 60_ = 7.56, *P* < 0.001). In the sham group, IOD values of SYP immunohistochemical staining were maintained at a stable level (3 d vs. 7 d vs. 14 d vs. 28 d, all *P* > 0.05 compared to each other). The expression of SYP in the model group was significantly higher than that of the sham group at 7 d (*P*=0.020), 14 d (*P* < 0.001), and 28 d (*P* < 0.001) after MCAO. Furthermore, SYP level was upregulated by TMP treatment at 7 d (*P*=0.016), 14 d (*P* < 0.001), and 28 d (*P*=0.002) after MCAO compared with the model group.  Figure 6 shows the SYP levels of three groups.

### 3.3. Effect of TMP on Expression of GAP-43

**Figure 7:**
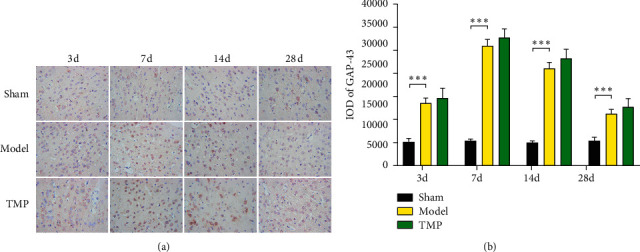
Identification of GAP-43 by immunohistochemical assay (× 400) in IP with time going on 3 d, 7 d, 14 d, and 28 d after MCAO. (a) Representative immunohistochemical images of GAP-43 expression. (b) Quantification of the GAP-43 levels through measuring the IOD values (mean ± SD, *n* = 6). ^*∗*^ indicates *P* < 0.05, ^*∗∗*^ indicates *P* < 0.01, and ^*∗∗∗*^ indicates *P* < 0.001.

The expression of GAP-43 was significantly affected by time (*F*_1.78, 26.74_ = 317.21, *P* < 0.001) and intervention (*F*_2, 15_ = 390.85, *P* < 0.001), and there was significant interaction (*F*_6, 45_ = 80.98, *P* < 0.001). Only weak GAP-43 immunostaining was observed in the sham group. In the control group, GAP-43 of IP appeared a sharply increase from 3 d to 7 d and reached the peak at 7 d; thereafter, the GAP-43 levels decreased gradually from 14 d to 28 d and still significantly was higher than that of the sham group (*P* < 0.001). In the TMP group, a similar pattern of time course was observed. However, TMP seemly could not regulate the GAP-43 levels at all time points (*P* > 0.05 compared to the model group).  Figure 7 shows the GAP-43 levels of three groups.

### 3.4. Effect of TMP on Changes in Synaptic Ultrastructures

**Figure 8:**
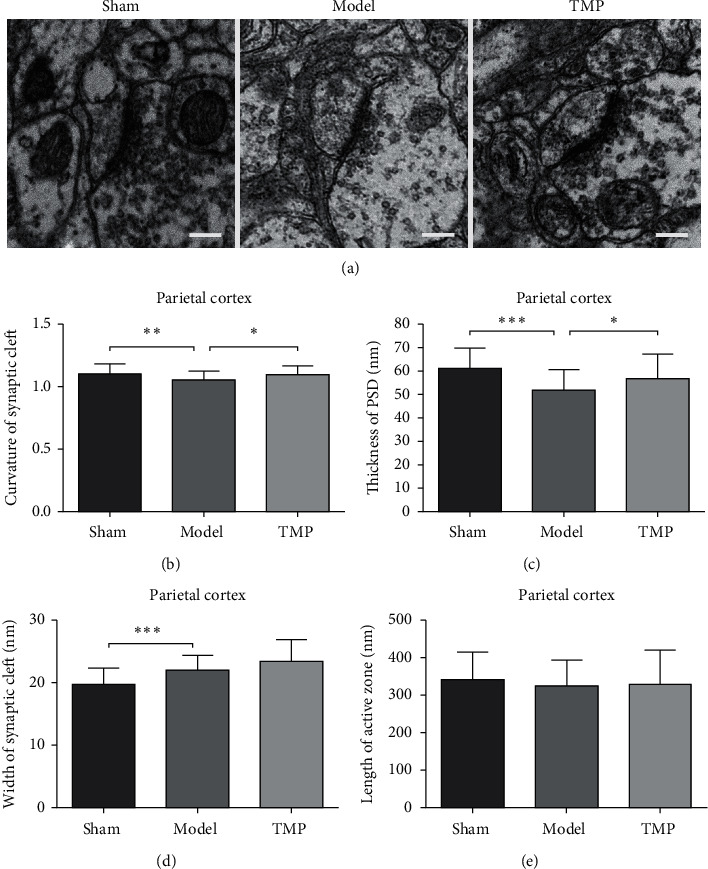
Synaptic ultrastructures in ischemia penumbra of parietal cortex at 28 d after MCAO. (a) Electromicrographs (× 10000) of synaptic ultrastructures (mean ± SD, *n* = 40 (40 graphs from 4 rats in each group)). (b) Comparison on curvature of synaptic interface. (c) Comparison on thickness of PSD. (d) Comparison on width of synaptic cleft. (e) Comparison on length of active zone. ^*∗*^ indicates *P* < 0.05, ^*∗∗*^ indicates *P* < 0.01, and ^*∗∗∗*^ indicates *P* < 0.001. Scale bar = 200 nm.

4 rats were sacrificed at 28 d after MCAO, and totally, 40 micrographs (10 per animal) of each group were taken for measurement. One-way ANOVA revealed that there were significant differences between three groups in curvature of synaptic interface (*F*2, 117 = 5.53, *P*=0.005), thickness of PSD (*F*2, 117 = 10.68, *P* < 0.001), and width of synaptic cleft (*F*2, 117 = 17.76, *P* < 0.001), whereas there is no difference in length of active zone (*F*2, 117 = 0.56, *P*=0.573). Compared with the sham group, the parameters of model group were partially changed with a less curved synaptic interface (*P*=0.007), a thinner PSD (*P* < 0.001), and a wider synaptic cleft (*P* < 0.001) at 28 d after MCAO. Some of these parameters could be modified by TMP to some extent. To be specific, synaptic interface curvature (*P*=0.029) and thickness of PSD (*P*=0.041) of TMP group were significantly higher compared with the sham group.  Figure 8 shows the synaptic ultrastructural parameters of three groups.

### 3.5. Correlation Analysis

**Figure 9:**
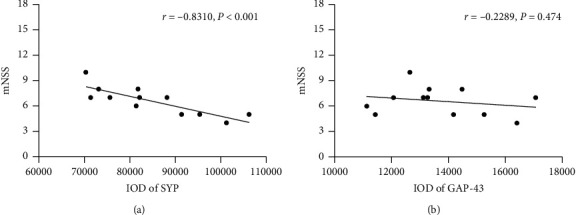
Pearson's correlation test. (a) Correlation between mNSS and SYP expression at 28 d after MCAO. (b) Correlation between mNSS and GAP-43 expression at 28 d after MCAO.

**Figure 10:**
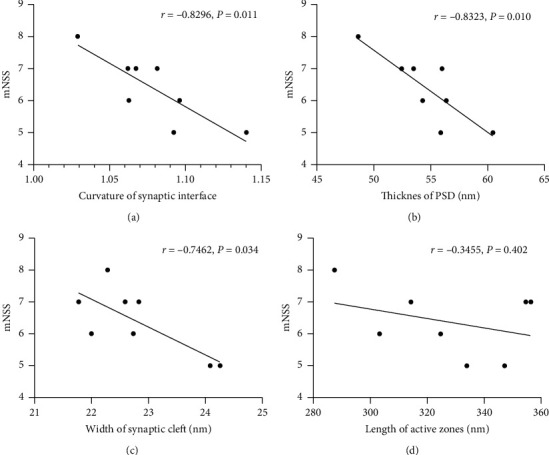
Pearson's correlation test between mNSS and synaptic ultrastructures at 28 d after MCAO. (a) Correlation between mNSS and curvature of synaptic interface. (b) Correlation between mNSS and thickness of PSD. (c) Correlation between mNSS and width of synaptic cleft. (d) Correlation between mNSS and length of active zone.

Pearson's correlation test showed that mNSS significantly correlated with SYP (*r* = −0.8310, *P* < 0.001), curvature of synaptic interface (*r* = −0.8296, *P*=0.011), thickness of PSD (*r* = −0.8323, *P*=0.010), and width of synaptic cleft (*r* = −0.7462, *P*=0.034) at 28 d after MCAO (Figures 9 and 10). However, there were no significant correlation between mNSS and GAP-43 (*r* = −0.2289, *P*=0.474) and length of the active zone (*r* = −0.3455, *P*=0.402) at 28 d after MCAO (Figures 9 and 10).

## 4. Discussion

The present study systematically evaluated the therapeutic neuroprotective effect of TMP in rats with ischemic brain injury induced by MCAO and revealed the positive role in the induction of neuroplasticity after ischemia reperfusion injury.

It is well known that the blood-brain barrier (BBB) comprised mainly of vascular endothelial cells and excluded from the brain approximately 100% of high-molecular-weight neurotherapeutics and more than 98% of all low-molecular-weight drugs [22]. TMP is one of the lucky members that can penetrate blood-brain barrier efficiently [23]. A majority of study focused on the effects of TMP at the acute stage after MCAO such as anti-platelet aggregation, anti-inflammatory, antiapoptosis, acceleration of vascular regeneration, inhibition of excitotoxicity, and promotion of endogenous neural stem cells [24], whereas our study presented the role of TMP on brain plasticity and may provide a longer time window of administration.

MNSS is a comprehensive score of motor, sensory, reflex, and balance tests, which is one of the most representative tools for the MCAO rat model [25]. The data of mNSS showed that neurological function of model rats displayed a spontaneous recovery, which may continue for many weeks at least. TMP improved mNSS scores at 14 d and 28 d after MCAO, suggesting TMP could ameliorate general health status of rats with MCAO.

SYP (38 KDa) is known as a reliable marker of synaptogenesis, whose expression level could reflect the density of synapses [26]. After ischemic injury, there is a series of changes (decrease in very early stage and increase later) in SYP level indeed [27]. However, in this study, the expression of SYP increased gradually from 3 d to 28 d after MCAO in IP, which indicated that there was recovery in number and function of synapses. Another study also showed the amount of immunoreactive synaptophysin was depressed until 3 d after ischemia reperfusion (may result from presynaptic degeneration and reduction in generation in the early stage) [28]. In our study, we observed the expression of SYP from 3 d after MCAO, and similar changes were not observed within 3 days. The improvement of SYP level was indicative of better synaptic plasticity and may be a possible mechanism contributing to observed functional improvement [29, 30]. Judging from the immunohistochemical data, we found that mNSS was highly correlated with SYP and TMP of 20 mg/kg and notably improved SYP level in IP at 7 d, 14 d, and 28 d after MCAO. These results may clarify one of the protective mechanisms of TMP to cerebral ischemia reperfusion. However, the specific regulatory pathway of TMP on synapses was unclear and may be related to brain-derived neurotrophic factor (BDNF)-tyrosine kinase B (TrkB) signaling. BDNF, a member of the neurotrophin family, and its specific receptor TrkB have been confirmed to play a pivotal role in establishment and maintenance of synapses [31]. Recent articles have demonstrated that TMP could effectively enhance expression of BDNF [14, 32], which may contribute to upregulation of SYP level although not investigated directly here.

GAP-43 (43 KDa) is a classical molecular marker for vertebrate axon growth and regeneration [33]. Upregulation of GAP-43 was observed in all nerve cells during axonal outgrowth and at early stages of synaptogenesis [34, 35]. Only a few expression of GAP-43 was observed in normal rat cerebral cortex, which was significantly elevated in IP cortex after MCAO [36]. GAP-43 immunostaining was used as a surrogate measure of axon growth and/or terminal sprouting in stroke models [37]. Immunohistochemical staining in the present study showed that GAP-43 remarkably increased at 3 d, continued until 7 d, and decreased from 14 d to 28 d after MCAO, which corresponded fairly closely with previous studies [38, 39]. Our data provided evidence of a dynamic process of axon growth and regeneration. However, even observed for nearly one month, no significant improvement of GAP-43 was observed with treatment of 20 mg/kg TMP, which may suggest that GAP-43 was not an effective target for TMP on cerebral ischemia reperfusion injury.

SYP and GAP-43 are two classical proteins participating in the synaptic structural plasticity [40]. Detecting synaptic proteins levels was helpful to show the regeneration after injury. To summarize the immunohistochemical staining data, a time-limited increase in structural plasticity happened in IP within one month after MCAO, including at least axonal growth and synaptogenesis. This finding was in agreement with previous work showing GAP-43 increased sharply earlier and SYP later subsequently and peaked at different time points (GAP-43 at 7 d and SYP at 28 d or later), indicating a hypothesis that there is axonal sprouting followed by synaptogenesis in IP after MCAO [41, 42].

There is an intimate relationship between synaptic plasticity and neural function after stroke [43]. Brain could regulate its function and structure constantly to cope with everyday external stimulates. Ischemia is a serious stimulate to brain apparently; therefore, it should come as no surprise that some compensatory changes take place in weeks and months after stroke [44]. Synaptic plasticity includes structural and functional plasticity. The morphology of synapses is the structural basis for synaptic function and plasticity [45]. In other words, morphological changes can cause functional changes and leads to alterations in synaptic transmission efficiency. TMP has been shown to promote SYP expression and facilitate synaptogenesis. The following four reliable parameters are usually measured in studies on synaptic ultrastructure: curvature of synaptic interface, thickness of PSD, width of synaptic cleft, and length of active zones [19, 46, 47]. In our study, compared to the sham group, the MCAO group displayed changes in the synaptic ultrastructures of a flatter interface, a thinner PSD, and a wider synaptic cleft at 28 d after MCAO. These changes could attenuate the transmitting efficacy and depress synaptic functional plasticity so as to function deficit. Although there was a natural recovery process of ultrastructures after ischemic injury [48], we still found visible differences of above three parameters, except the length of the active zones at 28 d MCAO. According to our results, TMP significantly reversed the changes in synaptic structural parameters (curvature of synaptic interface and thickness of PSD, which were correlated with mNSS, respectively) compared to the model group, elucidating that TMP's effect on improvement of neurological function may be associated with remodeling of synaptic ultrastructures. However, no obvious alterations were found in width of synaptic cleft and length of active zone. The present study provided evidence for TMP's role for participating in forming synaptic structural plasticity, particularly altering synaptic transmission efficiency.

## 5. Conclusion

In summary, our study suggests that TMP might regulate synaptic plasticity in a MCAO rat model. TMP significantly promoted neurological function of MCAO rats, which may be related to upregulation of SYP level and remodeling of synaptic ultrastructures.

## Data Availability

The data used to support the findings of this study are available from the corresponding author upon request.
